# A novel pathogenic variant in the carnitine transporter gene, *SLC22A5*, in association with metabolic carnitine deficiency and cardiomyopathy features

**DOI:** 10.1186/s12872-023-03676-z

**Published:** 2024-01-02

**Authors:** Amir Ghaffari Jolfayi, Niloofar Naderi, Serwa Ghasemi, Alireza Salmanipour, Sara Adimi, Majid Maleki, Samira Kalayinia

**Affiliations:** 1 Rajaie Cardiovascular Medical and Research Center, Iran University of Medical Sciences, Tehran, Iran; 2 Cardiogenetic Research Center, Rajaie Cardiovascular Medical and Research Center, Iran University of Medical Sciences, Tehran, Iran

**Keywords:** Primary carnitine deficiency, Cardiomyopathy, *SLC22A5*, Organic cation transporter 2, Whole-exome sequencing

## Abstract

**Background:**

Primary carnitine deficiency (PCD) denotes low carnitine levels with an autosomal recessive pattern of inheritance. Cardiomyopathy is the most common cardiac symptom in patients with PCD, and early diagnosis can prevent complications. Next-generation sequencing can identify genetic variants attributable to PCD efficiently.

**Objective:**

We aimed to detect the genetic cause of the early manifestations of hypertrophic cardiomyopathy and metabolic abnormalities in an Iranian family.

**Methods:**

We herein describe an 8-year-old boy with symptoms of weakness and lethargy diagnosed with PCD through clinical evaluations, lab tests, echocardiography, and cardiac magnetic resonance imaging. The candidate variant was confirmed through whole-exome sequencing, polymerase chain reaction, and direct Sanger sequencing. The binding efficacy of normal and mutant protein-ligand complexes were evaluated via structural modeling and docking studies.

**Results:**

Clinical evaluations, echocardiography, and cardiac magnetic resonance imaging findings revealed hypertrophic cardiomyopathy as a clinical presentation of PCD. Whole-exome sequencing identified a new homozygous variant, *SLC22A5* (NM_003060.4), c.821G > A: p.Trp274Ter, associated with carnitine transport. Docking analysis highlighted the impact of the variant on carnitine transport, further indicating its potential role in PCD development.

**Conclusions:**

The c.821G > A: p.Trp274Ter variant in *SLC22A5* potentially acted as a pathogenic factor by reducing the binding affinity of organic carnitine transporter type 2 proteins for carnitine. So, the c.821G > A variant may be associated with carnitine deficiency, metabolic abnormalities, and cardiomyopathic characteristics.

## Introduction

Primary carnitine deficiency (PCD) is characterized by low levels of carnitine in the body and is inherited as an autosomal recessive trait predominantly [1]. The incidence of PCD varies by ethnicity, with a frequency ranging from 1 in 142,000 in the United States [2] to 1 in 300 in the Faroe Islands [3]. The clinical manifestations of PCD vary from asymptomatic to sudden cardiac death [4]. Cardiomyopathy with or without skeletal muscle weakness starting from 1 to 4 years of age is the most frequent cardiac involvement in patients with PCD [5]. Among types of cardiomyopathies, dilated cardiomyopathy is the most common [6].

The *SLC22A5* gene on 5q31.1 is the sole gene responsible for systemic PCD and a member of the organic cation transporter family, which encodes organic carnitine transporter type 2 (OCTN2) [7]. *SLC22A5* spans 25,910 base pairs and contains 10 exons [8]. The final product of this gene is a 63-kilodalton protein composed of 557 amino acids, enhancing the uptake of carnitine [9]. *SLC22A5* encodes the OCTN2 transporter, responsible for carnitine uptake into cells. This process is crucial for transporting long-chain fatty acids into mitochondria for energy production. Various mutations ranging from missense and nonsense to splice-site mutations and deletions can be found in the *SLC22A5* gene, leading to the impaired function or expression of the OCTN2 transporter. The consequence is diminished cellular carnitine transport, which manifests in metabolic crises or cardiomyopathy due to disrupted fatty acid oxidation. The prevalence of specific mutations can vary across populations [10].

While PCD can induce dangerous complications and even death, early diagnosis and treatment can prevent many complications. New diagnostic techniques, including next-generation sequencing, contribute to advances in diagnosing hereditary heart diseases and identifying new genes related to those conditions [11]. Next-generation sequencing helps investigate the whole genome or exome to identify the genetic cause of diseases [12]. In the present study, we detected a novel pathogenic variant, *SLC22A5* (NM_003060.4), c.821G > A: p.Trp274Ter, in an Iranian family with early manifestations of hypertrophic cardiomyopathy and metabolic abnormalities, corresponding to the clinical and paraclinical symptoms of PCD. Although hypertrophic cardiomyopathy manifests in some patients with PCD, its precise etiology needs elucidation [13, 14]. Further in-depth information concerning the different variants of the genes involved in PCD can expedite its diagnosis and prevention.

## Methods

### Family recruitment and clinical evaluation

**Fig. 1:**
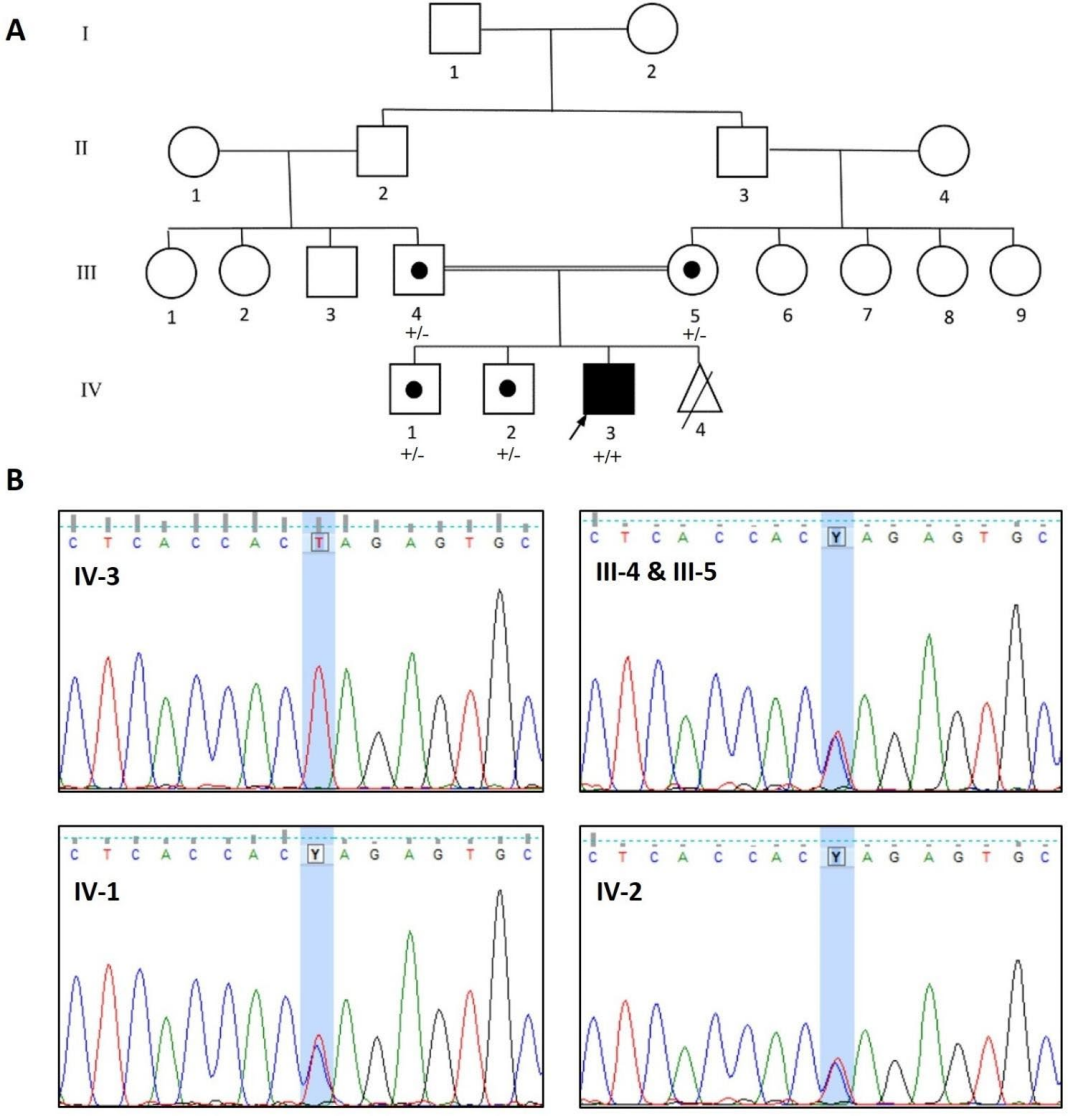
**A**: The image illustrates the pedigree of the family with primary carnitine deficiency (PCD). The proband (IV-3), indicated by the arrow, is the case of the study. **B**: The image presents the chromatogram of the change position. The individual marked with a black dot is a homozygous carrier. + sign: the reference allele; − sign: the mutant allele

The proband in the current case study was an 8-year-old boy (Fig. 1A: IV-3) referred to Rajaie Cardiovascular Medical and Research Center, affiliated with Iran University of Medical Sciences, Tehran, Iran, with complaints of weakness and lethargy. The patient’s consanguineous parents (first cousins) had no cardiomyopathy or metabolic disorders. The father and mother were 45 and 34 years of age, respectively. The boy was the third offspring and had 2 brothers, aged 26 and 19, without cardiac or metabolic diseases. The mother had a miscarriage for unknown reasons in the second month of pregnancy. The proband’s symptoms started 2 weeks prior to his hospital admission. The initial evaluation raised suspicions of glucose storage disease due to hypotonia and hepatomegaly. During the patient’s hospital stay, his weakness and lethargy responded to the prescription of bicarbonate and carnitine, and his symptoms disappeared gradually. The response to carnitine therapy bolstered the evidence of carnitine deficiency. The ammonia level was 155 µmol/L, indicating hyperammonemia, a consistent finding in patients with PCD. The boy underwent echocardiography, cardiac magnetic resonance imaging, and genetic testing for the confirmation of the diagnosis.

### Sampling and whole-exome sequencing (WES)

Informed consent was obtained from the proband and all his available family members, who subsequently provided peripheral blood samples. Genomic DNA was extracted from the peripheral blood via standard salting-out methods, following the manufacturer’s protocol. WES was performed on the proband (Fig. 1A: IV-3). Exome capture was conducted with an Agilent SureSelect All Exon V6 kit, and library sequencing was performed with the Illumina HiSeq 6000. An in-house setup bioinformatics pipeline, including quality checks and primary filtering of reads, aligning the reads to the human reference genome (GRCh37/hg19), and calling and annotating the variants, was applied. All variants with minor allele frequencies below 1% in the 1000 Genomes Project, the Genome Aggregation Database (gnomAD), and Iranome were considered.

Mutations that were regarded as likely pathogenic or pathogenic in ClinVar and the Human Gene Mutation Database (HGMD) and had a related phenotype were considered priorities. The remaining variants were subjected to bioinformatics analysis via online tools, such as MutationTaster, Polymorphism Phenotyping v2 (PolyPhen-2), Protein Variation Effect Analyzer (PROVEAN), Sorting Intolerant From Tolerant (SIFT), and Combined Annotation-Dependent Depletion (CADD), to predict the effects of the variants on the structure and function of the protein. Further, ENTPRISE-X was employed to predict the consensus of nonsense variants. The variants predicted as pathogenic by most of the tools were selected for segregation analysis. The standards of the American College of Medical Genetics and Genomics (ACMG) were applied to interpret the variants. Finally, the candidate variant was subjected to confirmation and segregation analysis.

### Polymerase chain reaction (PCR) and Sanger sequencing

Primer sequences surrounding the candidate variant were designed using the Primer3 (v. 0.4.0) server. PCR was performed with the SimpliAmp Thermal Cycler (Thermo Fisher Scientific) using 10 pmol/L of primers (forward primer: TATGGCGCCTTGGTCTTAGTA and reverse primer: TCAGCACACAGCCAGAACT), 100 ng of DNA, 200 mmol/L of dNTP, 1.5 mmol/L of MgCl2, and 1 U of Taq DNA polymerase (Amplicon). The PCR schedule was incubation at 95 °C for 5 min, followed by 35 cycles (40 s at 95 °C, 30 s at 59 °C, and 30 s at 72 °C). Subsequently, the PCR products were sequenced on the ABI Sequencer 3500XL PE (Applied Biosystems, USA) and the Codon Code Aligner (v. 7.1.2) (CodonCode Corp, USA).

### Structural modeling and docking study

The 3D structure of the human OCTN2 protein (SLC22A5) was not available in the protein data bank (PDB: http://www.rcsb.org/pdb). Consequently, the protein sequence (FASTA format) was downloaded from the National Center for Biotechnology Information (NCBI) database (https://www.ncbi.nlm.nih.gov/), and the 3D structure was constructed with AlphaFold2 using the MMseqs2 server [15]. For the generation of the 3D structures in the AlphaFold2 server, each protein sequence (the wild type and mutant [p.Trp274Ter]) was loaded in FASTA format into the AlphaFold2 server. Next, molecular modeling was initiated to generate the PDB format of each protein. The 3D structure of the L-carnitine molecule (vitamin BT, PubChem CID: 10,917) was accessed from the PubChem database (https://pubchem.ncbi.nlm.nih.gov/) and processed to a PDB file format. The structures of the proteins were corrected with ViewerLite (v.5), and polar hydrogens were added. Further, the YASARA Energy Minimization Server was utilized to minimize energy during molecular docking (http://www.yasara.org/minimizationserver.htm) [16]. The 3D formation of the compounds was imported as an SCE file into the YASARA View (v.20.12.24) to deliver low-energy structures of the compounds and was then saved in a PDB file format. Molecular docking was performed with AutoDock Vina to evaluate the binding efficacy of normal and mutant protein-ligand complexes [17, 18]. Post-docking analyses were visualized using PyMOL (v.2.5.2) and LigPlus+ (v.2.2.4), [19] providing details of the size and location of the binding sites and the hydrogen-bond interaction of the docked ligand in various confirmations.

### Ethical considerations

The study complies with the Declaration of Helsinki. Ethical approval was obtained from the Ethics Committees of Rajaie Cardiovascular Medical and Research Center, affiliated with Iran University of Medical Sciences, Tehran, Iran (IR.RHC.REC.1402.004). Written informed consent was obtained from the participants, who were ensured of their right to withdraw from the study at any point with no negative implications.

## Results

### Echocardiography and Cardiac magnetic resonance imaging findings

**Fig. 2:**
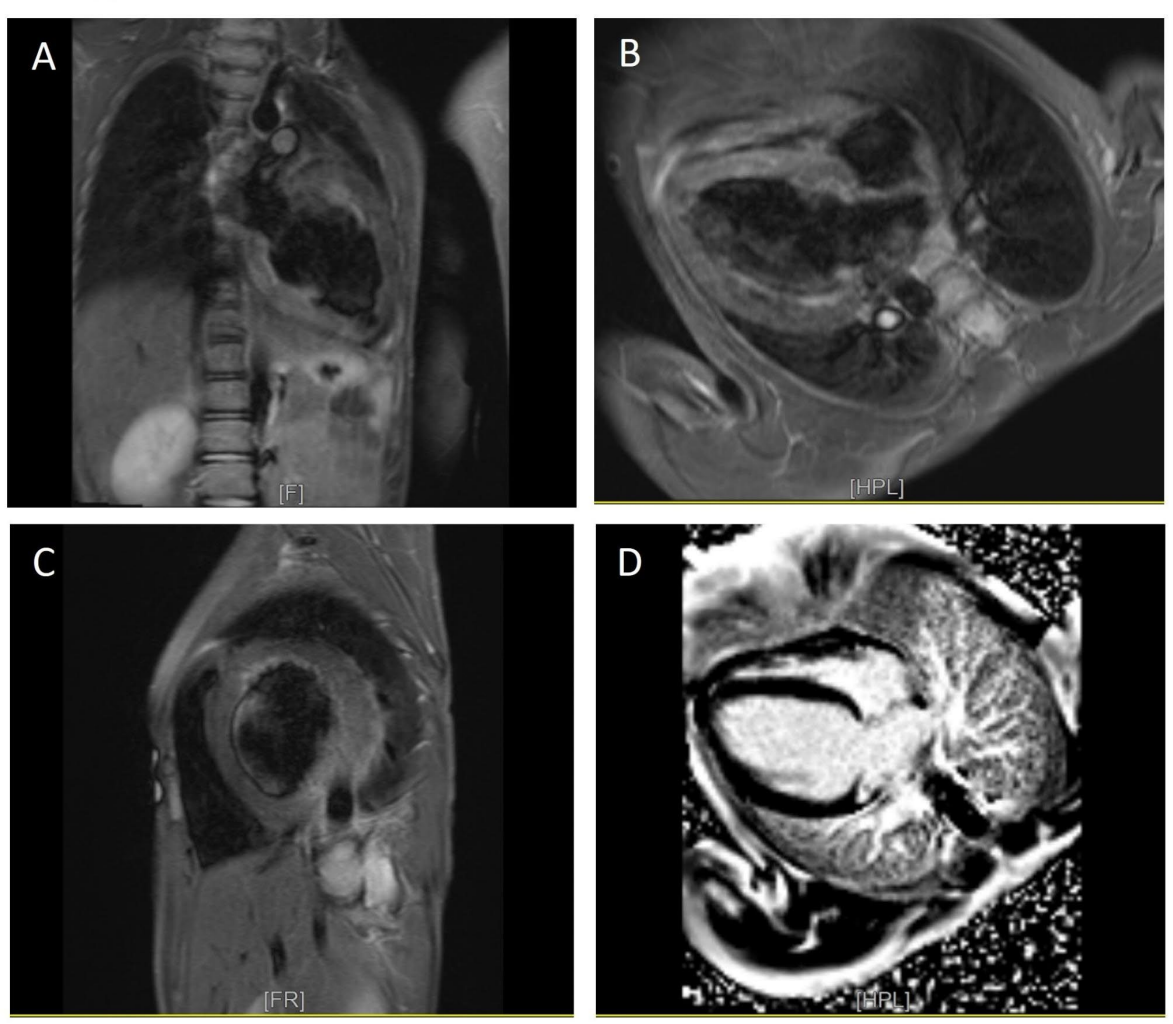
The image shows the proband’s cardiac magnetic resonance imaging. **A, B, and C**: They present 2- and 4-chamber views and short-axis short tau inversion recovery (STIR) images, respectively. Severe left ventricular enlargement is observed. **D**: The 4-chamber phase-sensitive inversion recovery (PSIR) with a 10-minute delay demonstrates no abnormal enhancement. Increased signal intensity adjacent to the subendocardial muscles in the STIR images is secondary to a significantly reduced ejection fraction and subsequent blood stasis

Echocardiography revealed mild left and right atrial enlargement, mild to severe left ventricular enlargement, mild left ventricular hypertrophy, left ventricular ejection fraction of 20%, severe right ventricular failure, tricuspid valve regurgitation, tricuspid annular plane systolic excursion of about 17 mm, and no atrial or ventricular septal defect and patent ductus arteriosus, favoring hypertrophic cardiomyopathy, an expected finding in carnitine deficiency. Cardiac magnetic resonance imaging showed cardiomegaly primarily due to left ventricular enlargement, increased left ventricular myocardial mass (mass index = 137 g/m^2^), left ventricular dyssynchrony, a severely reduced ejection fraction (≈ 17%), a cardiac index of 4 L/min/m^2^, moderate right ventricular enlargement without hypertrophy, a severely reduced systolic function (ejection fraction ≈ 24%), a right cardiac index of 2.9 L/min/m^2^, and no evidence of myocardial edema or enhancement (Fig. 2).

### Genetic findings

WES detected a novel pathogenic variant, c.821G > A: p.Trp274Ter, in a homozygous state in exon 4 of the carnitine transporter gene, *SLC22A5* (NM_003060). The identified variant was confirmed by Sanger sequencing. The variant was segregated in the pedigree with an autosomal recessive pattern. In other words, his healthy parents and 2 brothers had the heterozygous form of the variant (Fig. 1B). According to the ACMG/AMP guidelines (PVS1, PM2, and PM3), the identified variant was pathogenic. This variant has not been reported in any databases, and it appears to be the primary cause of PCD with hypertrophic cardiomyopathy manifestation in this pedigree.

### Modeling and Docking

**Fig. 3:**
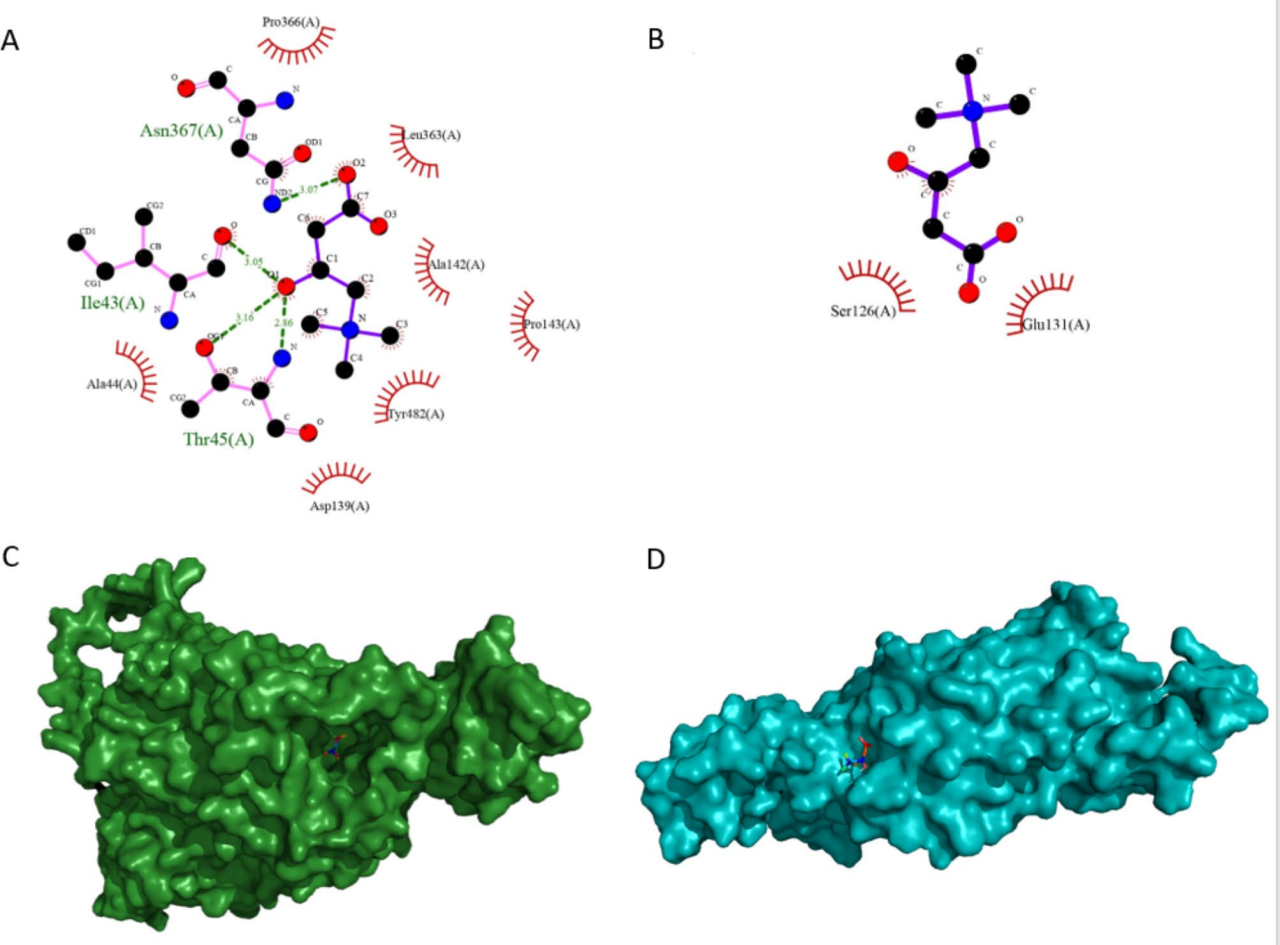
The images illustrate **(A)** the interaction between the wild-type protein and L-carnitine, **(B)** the interaction between the mutant protein and L-carnitine, **(C)** the 3D structure of normal organic carnitine transporter type 2 (OCTN2) (green) in interaction with L-carnitine, and **(D)** the 3D structure of the mutant OCTN2 (blue) in interaction with L-carnitine

The best models of human OCTN2 (normal and p.Trp274Ter) were downloaded from AlphaFold2 with predicted local distance difference test (pLDDT) scores of 85.7 and 90.2. The pLDDT score (0-100) indicates the identity of the reproduced model with the reference protein structure. Docking analysis was performed on OCTN2-ligand compounds in mutated and wild-type modes with root-mean-square deviations of 0 and 2.413 and docking scores of − 3.7 and − 4.4. The OCTN2 wild-type ligand compounds had a binding energy of − 4.4 kcal/mol and 4 hydrogen bonds between L-carnitine and Ile43, Thr45, and Asn367. The OCTN2 mutant ligand compounds (− 3.7 kcal/mol) had no connection with each other (Fig. 3 [A and B]). The buried surface area for the normal and mutant OCTN2-ligand compounds with L-carnitine is depicted in Fig. 3 (C and D).

## Discussion

An 8-year-old boy was referred to us with a metabolic disorder and cardiomyopathic features. Paraclinical examinations and laboratory biochemical tests were compatible with carnitine deficiency. To confirm the diagnosis, we performed WES and detected a novel pathogenic variant in *SLC22A5* (NM_003060.4), c.821G > A: p.Trp274Ter. This stop-gain variant led to an interruption in OCTN2 synthesis in the 274th amino acid of the peptide, and our bioinformatics analysis revealed no affinity between the transporter and carnitine as its ligand. Indeed, our docking results suggested that the truncated OCTN2 protein had no affinity with carnitine; therefore, the mutant *SLC22A5* (p.Trp274Ter) had significantly reduced carnitine transport activity compared with the wild-type *SLC22A5*.

The clinical features of *SLC22A5* variants and carnitine deficiency can vary widely. The reported variants in *SLC22A5* associated with carnitine deficiency phenotypes are presented in Table 1. In some cases, affected individuals may be asymptomatic, whereas others may present with life-threatening metabolic crises. The diagnosis of carnitine deficiency is based on clinical presentation, biochemical testing, and genetic analysis, with early diagnosis playing a crucial role in preventing side effects [20]. Spiekerkoetter et al. [21] concluded that patients with a similar genotype had different ages of onset and various types of clinical manifestations, including weakness, cardiac presentations, and metabolic abnormalities. Even siblings who possess the same variant in the *SLC22A5* gene have different onset ages and progressions [22]. *SLC22A5* stop-gain pathogenic variants are associated with carnitine deficiency or cardiomyopathy as the only clinical phenotype without metabolic abnormalities [23]. The primary treatment for carnitine deficiency is carnitine supplementation, [5] and affected individuals may require treatment for cardiomyopathy, liver dysfunction, and other complications [24]. Early diagnosis with precise diagnostic tools and prompt treatment with carnitine supplementation can improve outcomes and prevent complications; however, in severe cases, the disorder can give rise to life-threatening metabolic crises and lifelong disorders [24]. Carnitine deficiency, particularly PCD, is associated with cardiomyopathy, specifically dilated cardiomyopathy, as the most common type linked to PCD. In patients with PCD, dilated cardiomyopathy is more frequent, [25] but it does not mean that other cardiomyopathies do not exist in these patients. Hypertrophic cardiomyopathy is less commonly associated with PCD than dilated cardiomyopathy. In hypertrophic cardiomyopathy, the heart muscle thickens, obstructing blood flow and causing cardiac dysfunction [26].

**Table 1:** The reported variants in SLC22A5 associated with Carnitine deficiency phenotypes

No.	Nucleotide change	Amino acid change	dbSNP	CADD	ACMG	Mutation Type	Ref.
1	c.34G > A	p.Gly12Ser	rs139203363	28.3	LP	Missense	[1]
2	c.3G > T	p.Met1Ile	rs121908892	23.4	LP	Missense	[2]
3	c.12 C > G	p.Tyr4Ter	rs72552722	42	P	Nonsense	[3]
4	c.43G > T	p.Gly15Trp	rs267607052	32	P	Missense	[4]
5	c.47 C > T	p.Pro16Leu	-	21.5	LP	-	[5]
6	c.51 C > G	p.Phe17Leu	rs11568520	24.6	P	Missense	[6]
7	c.56G > C	p.Arg19Pro	rs72552723	25.0	P	Missense	[3]
8	c.77G > A	p.Ser26Asn	rs772578415	22.9	P	Missense	[7]
9	c.83G > T	p.Ser28Ile	rs72552724	25.0	P	Missense	[8]
10	c.95 A > G	p.Asn32Ser	rs72552725	24.7	P	Missense	[9]
11	c.131 C > T	p.Ala44Val	rs199689597	7.4	P	Missense	[10]
12	c.137 C > T	p.Pro46Leu	rs377767445	27.8	P	Missense	[5]
13	c.136 C > T	p.Pro46Ser	rs202088921	26.3	P	Missense	[11]
14	c.149G > A	p.Cys50Tyr	-	29.2	LP	-	[5]
15	c.185G > A	p.Trp62Ter	rs1554085942	42	P	Nonsense	[12]
16	c.196 A > C	p.Thr66Pro	-	24.0	VUS	Missense	[1]
17	c.224G > C	p.Arg75Pro	rs757711838	20.3	VUS	-	[1]
18	c.248G > T	p.Arg83Leu	rs72552726	31	P	Missense	[13]
19	c.278 C > G	p.Ser93Trp	rs386134190	31	LP	-	[5]
20	c.287G > C	p.Gly96Ala	rs377767450	27.8	VUS	Missense	[1]
21	c.338G > A	p.Cys113Tyr	rs727504159	32	P	Missense	[14]
22	c.344 A > G	p.Asp115Gly	rs386134192	33	VUS	Missense	[5]
23	c.350G > A	p.Trp117Ter	-	44	P	Nonsense	[1]
24	c.364G > T	p.Asp122Tyr	rs201082652	33	P	Missense	[1]
25	c.368T > G	p.Val123Gly	rs748605096	24.3	VUS	Missense	[1]
26	c.395G > A	p.Trp132Ter	rs886041277	51	P	Nonsense	[15]
27	c.420G > A	p.Trp140Ter	-	41	P	Nonsense	[5]
28	c.424G > T	p.Ala142Ser	rs151231558	14.5	P	Missense	[16]
29	c.428 C > T	p.Pro143Leu	rs1178584184	25.4	P	Missense	[6]
30	c.447 C > G	p.Phe149Leu	rs780989844	22.8	VUS	Missense	[17]
31	c.506G > A	p.Arg169Gln	rs121908889	31	P	Missense	[18]
32	c.506G > C	p.Arg169Pro	rs121908889	31	LP	Missense	[5]
33	c.505 C > T	p.Arg169Trp	rs121908890	29.6	P	Missense	[3]
34	c.523G > A	p.Val175Met	rs781721860	16.7	VUS	Missense	[5]
35	c.529 A > G	p.Met177Val	rs145068530	22.3	LP	Missense	[1]
36	c.535 A > T	p.Met179Leu	rs386134196	16.2	VUS	Missense	[19]
37	c.557T > C	p.Leu186Pro	rs386134197	25.8	LP	Missense	[1]
38	c.614T > G	p.Met205Arg	rs796052033	26.4	P	Missense	[5]
39	c.629 A > G	p.Asn210Ser	rs386134198	23.1	P	Missense	[5]
40	c.632 A > G	p.Tyr211Cys	rs121908888	29.3	P	Missense	[20]
41	c.641 C > T	p.Ala214Val	rs386134199	28.2	LP	Missense	[4]
42	c.674 C > T	p.Ser225Leu	rs386134205	24.3	LP	-	[5]
43	c.680G > A	p.Arg227His	rs185551386	32	P	-	[1]
44	c.688T > C	p.Phe230Leu	rs756650860	28.6	P	Missense	[1]
45	c.692 C > T	p.Ser231Phe	rs386134206	24.2	VUS	Missense	[5]
46	c.695 C > T	p.Thr232Met	rs114269482	27.4	P	Missense	[2]
47	c.700G > C	p.Gly234Arg	rs1457258524	28.3	LP	-	[6]
48	c.718G > A	p.Ala240Thr	-	22.6	LP	-	[1]
49	c.725G > T	p.Gly242Val	rs72552728	28.3	LP	Missense	[3]
50	c.740 C > G	p.Pro247Arg	-	26.7	P	Missense	[5]
51	c.761G > A	p.Arg254Gln	rs200699819	31	P	Missense	[17]
52	c.760 C > T	p.Arg254Ter	rs121908893	35	P	Nonsense	[21]
53	c.769 C > T	p.Arg257Trp	rs386134203	25.5	LP	Missense	[1]
54	c.791 C > G	p.Thr264Arg	rs201262157	24.8	P	Missense	[1]
55	c.797 C > T	p.Pro266Leu	rs538372785	24.1	P	Missense	[22]
56	c.825G > A	p.Trp275Ter	rs386134207	49	P	Nonsense	[2]
57	c.839 C > T	p.Ser280Phe	rs386134208	28.7	P	Missense	[23]
58	c.845G > A	p.Arg282Gln	rs386134210	32	P	Missense	[16]
59	c.844 C > T	p.Arg282Ter	rs121908886	39	P	Nonsense	[24]
60	c.847T > C	p.Trp283Arg	rs72552729	30	LP	Missense	[16]
61	c.849G > T	p.Trp283Cys	rs386134211	34	LP	-	[19]
62	c.865 C > T	p.Arg289Ter	rs386134212	36	P	Nonsense	[2]
63	c.902 C > A	p.Ala301Asp	rs72552730	26.9	LP	Missense	[25]
64	c.976 C > T	p.Gln326Ter	rs1047810495	37	P	Nonsense	[26]
65	c.1051T > C	p.Trp351Arg	rs68018207	33	VUS	Missense	[25]
66	c.1064 C > T	p.Ser355Leu	rs1385634398	24.1	VUS	-	[1]
67	c.1072T > A	p.Tyr358Asn	rs61731073	31	P	Missense	[1]
68	c.1085 C > T	p.Ser362Leu	rs886042092	27.1	VUS	Missense	[6]
69	c.1088T > C	p.Leu363Pro	rs386134214	29.5	LP	Missense	[27]
70	c.1139 C > T	p.Ala380Val	rs746187344	23.5	LP	Missense	[22]
71	c.1159T > C	p.Tyr387His	-	27.0	LP	Missense	[17]
72	c.1188T > G	p.Tyr396Ter	rs1057519051	33	P	Nonsense	[6]
73	c.1193 C > T	p.Pro398Leu	rs144547521	25.7	P	Missense	[16]
74	c.1196G > A	p.Arg399Gln	rs121908891	29.8	P	Missense	[3]
75	c.1195 C > T	p.Arg399Trp	rs267607054	27.4	P	Missense	[4]
76	c.1232G > T	p.Gly411Val	-	28.0	VUS	Missense	[28]
77	c.1313G > A	p.Gly438Glu	rs1580894230	26.8	P	Missense	[29]
78	c.1316T > G	p.Val439Gly	-	27.4	LP	-	[5]
79	c.1319 C > T	p.Thr440Met	rs72552732	25.6	P	Missense	[9]
80	c.1327T > G	p.Phe443Val	-	24.2	LP	-	[1]
81	c.1336G > T	p.Val446Phe	rs72552733	25.8	P	Missense	[30]
82	c.1340 A > G	p.Tyr447Cys	rs386134218	28.1	P	Missense	[8]
83	c.1342G > T	p.Val448Leu	rs386134219	23.3	LP	-	[5]
84	c.1345T > G	p.Tyr449Asp	rs11568514	27.3	LP	Missense	[2]
85	c.1354G > A	p.Glu452Lys	rs72552734	29.6	P	Missense	[25]
86	c.1364 C > G	p.Pro455Arg	rs1408166345	25.9	P	Missense	[1]
87	c.1385G > A	p.Gly462Asp	-	25.6	LP	Missense	[31]
88	c.1385G > T	p.Gly462Val	-	23.6	LP	Missense	[5]
89	c.1400 C > G	p.Ser467Cys	rs60376624	25.7	P	Missense	[19]
90	c.1403 C > G	p.Thr468Arg	rs386134221	20.7	P	Missense	[9]
91	c.1409 C > T	p.Ser470Phe	rs386134222	26.6	P	Missense	[9]
92	c.1412G > A	p.Arg471His	rs386134223	31	P	Missense	[32]
93	c.1412G > C	p.Arg471Pro	-	32	LP	Missense	[7]
94	c.1411 C > T	p.Arg471Cys	rs749282641	27.0	P	Missense	[6]
95	c.1427T > G	p.Leu476Arg	-	26.6	LP	-	[33]
96	c.1433 C > T	p.Pro478Leu	rs72552735	27.7	LP	Missense	[34]
97	c.1458 C > G	p.Tyr486Ter	rs763224132	33	P	Nonsense	[35]
98	c.1463G > A	p.Arg488His	rs28383481	27.6	LP	Missense	[16]
99	c.1462 C > T	p.Arg488Cys	rs377216516	27	P	Missense	[11]
100	c.1520T > C	p.Leu507Ser	rs1157198543	28.9	LP	-	[1]
101	c.1645 C > T	p.Pro549Ser	rs11568525	18.2	B	Missense	[36]
102	c.393 + 5G > A	-	rs1554086029	24.2	VUS	Intron	[14]
103	c.394-16T > A	-	rs775097754	22.5	LP	Intron	[7]
104	c.497 + 1G > T	-	-	35	P	Splice donor variant	[14]
105	c.653-2 A > C	-	rs386134201	34	P	Splice donor variant	[5]
106	c.825-52G > A	-	rs1194929977	0.4	LP	Intron	[10]
107	c.1451-1G > A	-	rs386134224	33	P	Splice donor variant	[15]
108	c.67_69delTTC	p.Phe23del	rs377767444	37	P	Deletion	[9]
109	c.234_234delC	p.His79Thrfs*51	rs377767447	24.6	P	Deletion	[16]
110	c.235_238delCACA	p.His79Alafs*50	-	25	LP	Deletion	[37]
111	c.458_459delTG	p.Val153Alafs*41	rs386134195	32	P	Deletion	[2]
112	c.517_517delC	p.Leu173Cys fs*3	-	29.6	LP	Deletion	[14]
113	c.565_568delTTCT	p.Phe189Argfs*14	-	31	LP	Deletion	[5]
114	c.573_573delG	p.Asn192Ilefs*12	-	27.8	LP	Deletion	[1]
115	c.597_597delG	p.Phe200Leufs*4	-	29.8	LP	Deletion	[38]
116	c.745_748delTTTG	p.Phe249Leufs*14	-	31	LP	Deletion	[14]
117	c.806_806delT	p.Leu269Hisfs*27	rs386134204	23.5	P	Deletion	[39]
118	c.919_919delG	p.Val307Leufs*15	-	18	P	Deletion	[26]
119	c.1009_1009delA	p.Thr337Profs*12	rs386134213	28.3	LP	Deletion	[9]
120	c.1175_1177delTGC	-	-	28		Deletion	[5]
121	c.1303_1303delG	p.Gly435Alafs*24	rs386134217	33	P	Deletion	[24]
122	c.1304_1313delTGGGCAAGTT	p.Gly435Glufs*21	-	36		Deletion	[1]
123	c.1372_1372delG	p.Val458*	-	33		Deletion	[14]
124	c.4_5insC	p.Arg2Profs*136	rs377767443	28	P	Insertion	[15]
125	c.264_265insGGCTCGCCACC	p.Ile89Glyfs*45	-	36		Insertion	[3]
126	c.433_434insC	p.Ile146Asnfs*49	-	28.7		Insertion	[14]
127	c.1202_1203insA	p.Tyr401*	rs121908887	24	P	Insertion	[24]
128	c.1316_1317insT	p.Thr440Hisfs*83	-	28		Insertion	[40]
129	c.1556_1559insACAC	p.Ile521Hisfs*3	rs386134225	31	P	Insertion	[11]
130	c.1324_1325delinsAT	p.Ala442Ile	rs267607053	38.2	P	Indels	[4]
131	c.1392_1409delinsCA	p.Val465Thrfs*29	rs386134220	35	LP	Indels	[5]

P: Pathogenic; LP: Likely Pathogenic; VUS: Uncertain Significance; B: Benign

Research on the *SLC22A5* gene is limited to patients with hypertrophic cardiomyopathy. Still, the literature contains studies on the coincidence of PCD and hypertrophic cardiomyopathy in *SLC22A5* variants. Lahrouchi et al. [13] conducted WES on the members of a consanguineous family with a history of childhood hypertrophic cardiomyopathy and sudden cardiac death and reported a homozygous stop variant in the *SLC22A5* gene, typically associated with PCD, as the probable genetic culprit. Ino et al. [27] retrospectively assessed 11 children with abnormal carnitine metabolism and cardiomyopathy to determine the prognosis of cardiomyopathy correlated with hypocarnitinemia. The children exhibited various carnitine-related disorders: 6 of them showed hypertrophic cardiomyopathy, and the rest suffered from dilated cardiomyopathy. Echocardiography revealed inconsistent left ventricular function and wall thickness. In histologic evaluations, Ino and colleagues observed significant lipid buildup within enlarged heart cells. The authors concluded that carnitine supplementation conferred echocardiographic improvements for 6 out of 8 treated patients over periods of 3 months to 2 years. García-Vielma et al. [28] sought to identify genetic variants related to hypertrophic cardiomyopathy in Mexican patients. The authors maintained that while most global research primarily pointed to genes like *MHY7* and *MYBPC3*, there was a dearth of data on other genes. They evaluated 37 patients with heart disease and sudden death and focused on 168 genes. Among the identified pathogenic variants, the *SLC22A5* gene was an affected gene, along with others like *MYH7* and *MYBPC3*. Notably, 5 variants, including potentially the one in *SLC22A5*, had not been previously documented in public databases. In a case report, Deswal et al. [29] described a 9-month-old boy suffering from hypertrophic cardiomyopathy, significant hepatomegaly, and jaundice with extremely low free carnitine levels. Genetic analysis identified compound heterozygous mutations in the *SLC22A5* gene. One of these mutations had been previously reported, while the other was a novel frame-shift mutation. After 3 months of oral carnitine supplementation, the patient showed significant improvement, including an ejection fraction of 75% and regular liver size and enzymes. It is noteworthy that the proband in our study had no other hepatic-related signs. Mutlu-Albayrak et al. [41] described a 9-year-old boy with a dysmorphic appearance and hypertrophic cardiomyopathy. Both the patient and his 4-year-old sister, who also had cardiomyopathy and fatigue, were diagnosed with a carnitine uptake defect through tandem mass spectrometry. In addition, the patient’s other sister died suddenly at 19 months. Genetic sequencing identified a new mutation in the *SLC22A5* gene in both siblings, responsible for their carnitine uptake defect. The parents were found to be carriers of this mutation.

In conclusion, the paucity of information on PCD patients with hypertrophic cardiomyopathy involvement in *SLC22A5* variants warrants future studies to reveal the role of this gene in the development of coincident PCD and hypertrophic cardiomyopathy.

### Limitations

The findings of the present study should be interpreted in light of its limitations. Firstly, structural modeling and docking studies, albeit informative, cannot replace direct functional assays since they render the actual effects of the mutation on carnitine transport function speculative until these impacts are verified by in vivo studies. Nonetheless, in vivo or clinical interventions are not feasible due to ethical or cost-benefit considerations. Secondly, we did not consider other potential comorbidities and environmental factors that might have influenced the observed clinical manifestations. Thirdly, it is essential to recognize the potential phenotypic variability since not every individual with the same mutation may display the same clinical features. Fourthly, a study focusing on an Iranian family raises questions about the generalizability of the results to other ethnic or racial groups. Finally, while WES is powerful, it concentrates only on exonic areas, possibly missing causative variants in other crucial genomic regions and creating a potential detection bias. Further studies are, therefore, required to elucidate the pathogenicity of the variant identified in the current investigation.

## Conclusions

The present study detected a novel genetic variant, *SLC22A5* (NM_003060.4), c.821G > A: p.Trp274Ter by WES and *in-silico* study demonstrated a lower affinity to carnitine. The variant likely caused the abnormal manifestations and lab tests in the studied 8-year-old boy with symptoms of weakness and lethargy.

## Data Availability

All data generated or analyzed during this study are included in this published article.
